# Dietary Fiber and Glucagon-Like Peptide-1 Receptor Agonists in Obesity Management: Converging Mechanisms, Interactions, and Strategies for Durable Weight Control

**DOI:** 10.1016/j.advnut.2026.100647

**Published:** 2026-05-07

**Authors:** Yuxin Wang, Jiaxin Liu, Kristin Verbeke, Naschla Gasaly Retamal, Renate Akkerman, Paul de Vos

**Affiliations:** 1 Centre for Healthy eating & Food innovation (HeFi), Sustainable Foods and Health group, Maastricht University, Venlo, The Netherlands; 2 Translational Research Center in Gastrointestinal Disorders, KU Leuven, Leuven, Belgium

**Keywords:** obesity, GLP-1 receptor agonists, dietary fiber, adverse effect management, long-term weight control, combination strategy

## Abstract

Glucagon-like peptide-1 receptor agonists (GLP-1RAs) have transformed the management of obesity by producing substantial and durable weight loss. However, gastrointestinal adverse effects, including nausea, vomiting, and constipation, are a common, dose-dependent, and frequent cause of discontinuation. Furthermore, weight regain is typical after drug withdrawal, reflecting the chronic and relapsing nature of obesity. Long-term adherence is essential but is often constrained by high cost, injection burden, and patient preference. Moreover, the consequences of chronic GLP-1 receptor activation on gut physiology, microbiota composition, and immune tolerance remain incompletely defined. In parallel, dietary fibers offer a physiological means of engaging the same gut–brain axis through microbial fermentation and the stimulation of endogenous GLP-1. Fibers deliver broad benefits because they strengthen gut barrier function, enrich short-chain fatty acids, and recalibrate immunity toward an anti-inflammatory state. Nevertheless, weight loss with fiber alone is typically more modest than with GLP-1RAs and depends on the type, dose, and duration of use. Tolerability can be limited by bloating or gas, particularly if intake is increased too rapidly. This review critically examines the convergence and divergence between GLP-1RAs and dietary fibers. We discuss their mechanistic overlaps in appetite control, metabolism, and immune modulation, and highlight potential interactions, such as altered fermentation dynamics during pharmacological slowing of gastric emptying and the potential for GLP-1R desensitization. We explore opportunities for fibers to mitigate GLP-1RA–related adverse effects, support bowel regularity, and stabilize the microbiota during treatment or after discontinuation. A pragmatic framework is raised to place dietary fiber and lifestyle measures as the foundation of care, reserve GLP-1RA therapy for the highest-risk individuals, and plan for fiber supplements once pharmacotherapy is reduced. Well-designed trials that combine GLP-1RAs with well-characterized fibers, include microbiome endpoints, and assess long-term outcomes are needed to optimize efficacy and reduce dependence on costly pharmacotherapy.

**Statement of Significance**

This review uniquely synthesized mechanisms linking GLP-1 receptor agonists and dietary fibers through the gut–brain axis, microbiota-derived metabolites, and immune modulation. It identified practical strategies based on their potential interactions for combination therapy to mitigate gastrointestinal adverse effects, improved durability after drug withdrawal, and proposed testable hypotheses for precision fiber selection during and after GLP-1RA therapy.

## Introduction

The global prevalence of obesity has risen markedly over recent decades, making it a major public health challenge that already affects >1 billion people worldwide and is projected to reach ∼2.16 billion by 2030 [1]. Obesity arises from complex interactions among obesogenic environments, poor dietary patterns, and insufficient physical activity, and is increasingly recognized as a chronic disease with multifactorial biological drivers [2,3]. Conventional management has largely relied on lifestyle modification, particularly caloric restriction and increased physical activity. However, these approaches are often difficult to sustain and frequently fail to achieve durable weight loss, in part because compensatory physiological adaptations increase appetite and reduce energy expenditure [4]. These observations highlight the need for therapeutic strategies, including dietary interventions, which target the underlying biological determinants of obesity.

Among the pathways involved in appetite and body weight regulation, the gut–brain axis has emerged as a central regulator of energy homeostasis [5]. The intestine is not only responsible for nutrient digestion and absorption but also functions as an endocrine and immune organ that integrates metabolic and immunological signals [6]. Within the intestinal epithelium, enteroendocrine cells, particularly L cells, sense luminal nutrients and secrete glucagon-like peptide-1 (GLP-1), an incretin hormone that suppresses appetite, delays gastric emptying, and enhances glucose-dependent insulin secretion [7]. A growing understanding of GLP-1 biology has led to 2 major therapeutic concepts in obesity management: pharmacological activation of the GLP-1 receptor (GLP-1R) and nutritional strategies that stimulate endogenous GLP-1 secretion [8,9].

GLP-1 receptor agonists (GLP-1RAs), such as liraglutide and semaglutide, have transformed obesity treatment by producing substantial weight loss and improving glycemic control. Their prolonged activity results from resistance to degradation by dipeptidyl peptidase-4 (DPP-4) and optimized pharmacokinetic properties [10]. Despite their clinical efficacy, important limitations remain. Gastrointestinal (GI) adverse effects, such as nausea, vomiting, diarrhea, and constipation, are common, particularly during treatment initiation and dose escalation, and may reduce adherence [11]. Clinical benefits generally require continuous therapy, with substantial weight regain frequently observed after discontinuation [12]. In addition, high costs, the burden of frequent injections, and uncertainties regarding the long-term impact of chronic GLP-1R activation on gut physiology, microbiota composition, and immune tolerance may further restrict broader use [13,14]. These limitations support interest in complementary nonpharmacological strategies that engage related gut-derived pathways.

Dietary fibers represent one such strategy. Fermentable fibers are metabolized by gut microorganisms into short-chain fatty acids (SCFAs), which can stimulate colonic L cells and promote endogenous GLP-1 secretion [[15], [16], [17]]. Beyond incretin modulation, dietary fibers enhance intestinal barrier integrity, promote the differentiation of regulatory T cells (Tregs), and mitigate metabolic endotoxemia [18]. Although the weight-loss effects of dietary fibers are generally modest and depend on fiber type, dose, and duration, their broader physiological benefits make them relevant to long-term weight management [19]. Because GLP-1RAs and dietary fibers converge on overlapping physiological pathways, they should be viewed not as competing strategies, but as potentially complementary ones. GLP-1RAs provide rapid and potent metabolic effects, whereas dietary fibers support microbial resilience, bowel regularity, and sustained GLP-1 production, which may facilitate the transition to weight maintenance as drug therapy is tapered [12,20,21]. At the same time, drug-induced slowing of gastric emptying could alter fiber tolerance and fermentation dynamics [22,23]. At present, such bidirectional interactions should be regarded as conceptual, hypothesis-generating frameworks rather than evidence-based treatment strategies.

This review critically evaluates pharmacological GLP-1RAs and dietary fibers as complementary strategies for obesity management, with emphasis on their convergent and divergent effects on metabolism, immunity, gut physiology, and the intestinal microbiota. It contrasts the potent receptor-mediated effects of GLP-1RAs with the indirect effects of dietary fibers through microbial fermentation and stimulation of endogenous GLP-1 secretion. Potential intersections and friction between these approaches are also discussed, including whether fiber supplementation may enhance tolerability and sustain long-term efficacy during or after GLP-1RA therapy.

## Methods

### Review approach and scope

This narrative review was written after a structured literature search in Google Scholar, Web of Science, and PubMed for studies relevant to obesity, GLP-1, GLP-1RAs, dietary fiber, SCFAs, gut microbiota, immunity, and gut physiology. Particular attention was given to literature relevant to the potential overlap, interaction, and therapeutic complementarity between GLP-1RAs and dietary fibers in obesity management. Additional articles were identified by screening the reference lists of relevant papers. Studies were selected according to their relevance to the scope of the review and their overall scientific quality.

### Selection criteria

Eligible sources included original research articles, clinical trials, systematic and comprehensive reviews, clinical guidelines, and relevant reports or recommendations, including documents from organizations such as the WHO, where appropriate. Animal and in vitro studies were also included when they provided important mechanistic insight. Because this was a narrative review, no formal systematic review protocol or risk-of-bias assessment was applied.

## Role of Dietary Fibers in Obesity and Weight Management

### Dietary fiber-induced GLP-1 secretion from intestinal L cells and its metabolic effects

**FIGURE 1:**
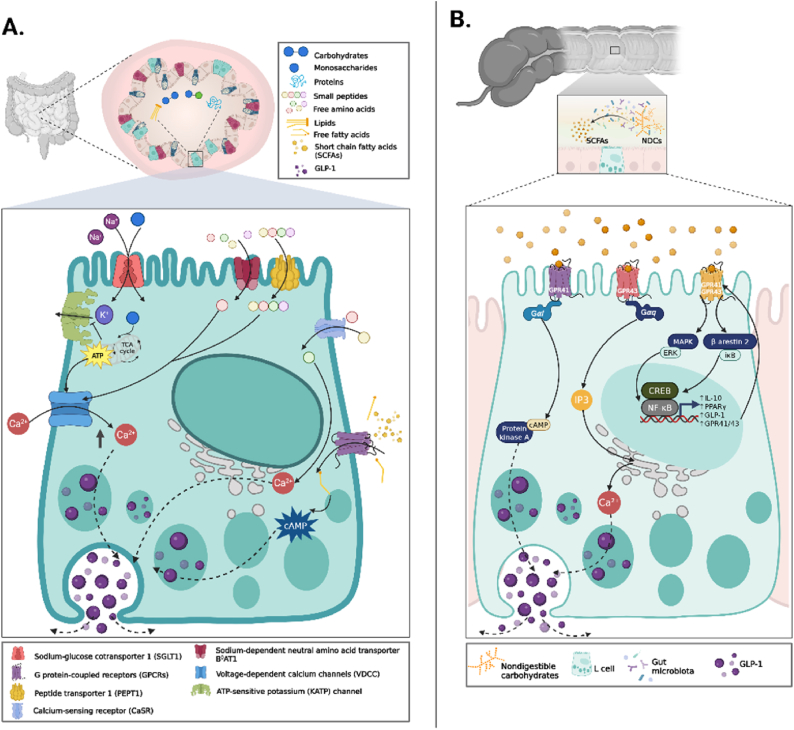
Dietary signals driving GLP-1 secretion from intestinal L cells. (A) How multiple nutrient classes converge to trigger GLP-1 secretion from enteroendocrine L cells. Carbohydrates are sensed via SGLT1-dependent glucose uptake, leading to ATP generation, KATP channel closure, and calcium influx that drives vesicle exocytosis. Longer-chain fatty acids stimulate GLP-1 release through GPR40/FFAR1 and GPR120/FFAR4 signaling, whereas SCFAs generated by microbial fermentation activate GPR41/FFAR3 and GPR43/FFAR2. Amino acids and dipeptides engage the CaSR and transporters such as PEPT1, inducing membrane depolarization. Collectively, these receptor- and transporter-mediated pathways orchestrate nutrient-dependent endocrine signaling that links diet composition to satiety, glycemic control, and gut–brain communication. (B) Microbial fermentation of dietary fibers generates SCFAs, such as acetate, propionate, and butyrate, which act on L-cell receptors FFAR2 and FFAR3 to promote GLP-1 secretion. SCFAs also serve as metabolic substrates for colonocytes, enhancing ATP production and membrane depolarization that facilitate calcium-dependent hormone release. Beyond endocrine signaling, SCFAs strengthen intestinal barrier integrity and promote anti-inflammatory IL-10 production, linking microbial metabolism to improved immune tolerance and metabolic health. (B) These parallel endocrine and immunometabolic effects of microbial fermentation. FFAR, free fatty acid receptor; GLP-1, glucagon-like peptide-1; MAPK, mitogen-activated protein kinase; NDCs, nondigestible carbohydrates; NF-κB, nuclear factor kappa B; PPAR, peroxisome proliferator-activated receptor; TCA, tricarboxylic acid cycle.

GLP-1 is predominantly secreted by enteroendocrine L cells. The density of these cells increases toward the distal regions of the GI tract, with the majority located in the distal ileum and colon, where they sustain a later phase of postprandial secretion [24]. However, GLP-1 concentrations rise within 15 min of a meal, indicating a substantial early contribution from L cells activated by nutrients in the small intestine [25,26]. A schematic overview of these GLP-1 nutrient-sensing pathways is shown in Figure 1. Carbohydrates, amino acids, and fatty acids likely act mainly in the small intestine, whereas SCFAs primarily stimulate GLP-1 secretion in the colon.

Dietary fibers, including resistant starch and other nondigestible carbohydrates (NDCs), undergo microbial fermentation in the colon to generate SCFAs such as acetate, propionate, and butyrate. These SCFAs act on L cells and increase the production of GLP-1. For example, in rats, dietary supplementation with 12% resistant starch increased plasma GLP-1 concentrations at all sampled time points across a 24-h period and improved oral glucose tolerance. These effects were accompanied by higher proglucagon expression in the caecum and colon, supporting the hypothesis that fermentation-derived SCFAs stimulate L-cell endocrine output and GLP-1 release [27]. SCFAs are sensed by enteroendocrine L cells via free fatty acid receptor (FFAR) FFAR2 (GPR43) and FFAR3 (GPR41). These receptors differ in G protein coupling: free fatty acid receptor 2 (FFAR2) engages Gαq/11 and Gαi/o, whereas FFAR3 predominantly couples to Gαi/o [28]. This enables SCFAs to activate parallel phospholipase C-dependent calcium and cAMP-sensitive pathways that converge on vesicle exocytosis [29]. Tolhurst et al. [30] showed that physiological mixtures of acetate, propionate, and butyrate rapidly increase intracellular Ca^2+^ and stimulate GLP-1 secretion in primary colonic cultures. Moreover, genetic deletion of either FFAR2 or FFAR3 attenuated incretin responses both in vitro and in vivo, reduced colonic GLP-1 content, and impaired glucose tolerance, underscoring the complementary roles of these receptors.

Receptor signaling, however, is not the only pathway through which SCFAs stimulate GLP-1 release. In an isolated vascularly perfused rat colon preparation, Christiansen et al. [31] showed that acetate and butyrate, but not propionate, promoted a significant GLP-1 secretion, but only under permissive intracellular cAMP conditions. This response disappeared when K_ATP channels were forced open with diazoxide, when voltage-dependent Ca^2+^ channels were blocked with nifedipine, or when oxidative phosphorylation was inhibited by 2,4-dinitrophenol. These findings indicate that colonocytes preferentially oxidize SCFAs to generate ATP, depolarize the membrane, and allow Ca^2+^ influx to trigger GLP-1 secretion [30,32,33]. Taken together, microbial fermentation converts dietary fibers into SCFAs that activate nutrient-sensing and metabolic pathways, thereby improving the secretion of GLP-1 from L cells. Accordingly, supplementing the diet with fermentable dietary fibers that increase the production of SCFAs may provide a strategy to potentiate endogenous GLP-1, improving metabolic health.

**FIGURE 2:**
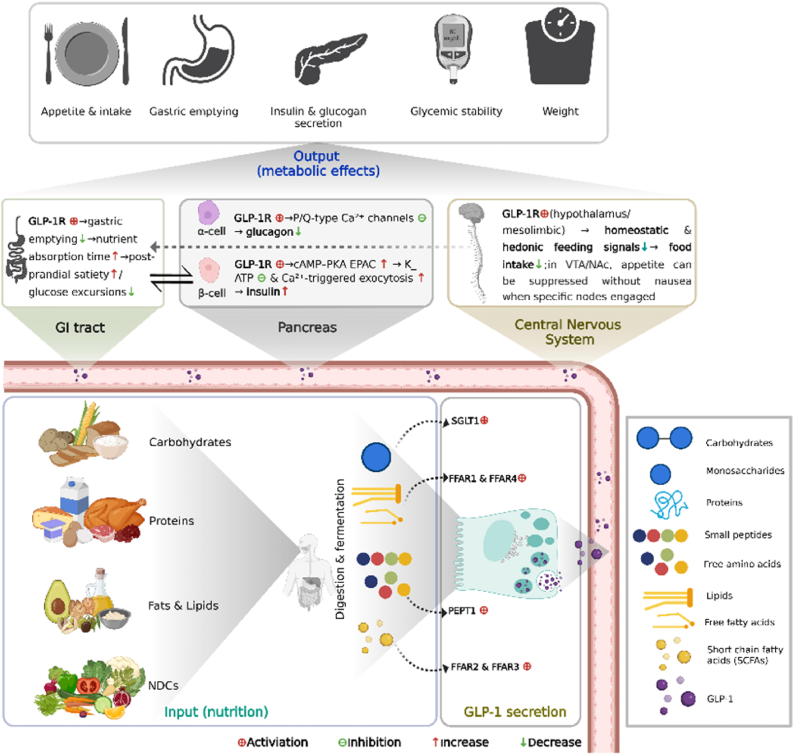
Nutrient sensing and GLP-1–mediated gut–brain–pancreas axis. The figure depicts the integration of intestinal nutrient sensing with systemic metabolic regulation through the gut–brain–pancreas axis. Upon nutrient ingestion, L-cell–derived GLP-1 activates vagal afferents and hypothalamic circuits that suppress appetite and enhance satiety. Concurrently, GLP-1 receptor activation in pancreatic *β*-cells amplifies glucose-stimulated insulin secretion, inhibits glucagon release from α-cells, and promotes *β*-cell proliferation and survival. Peripheral actions, including delayed gastric emptying and modulation of hepatic glucose output, collectively reinforce glycemic stability and reduce energy intake. This integrated axis illustrates the central role of GLP-1 as a hormonal coordinator of metabolic homeostasis. EPAC, exchange protein directly activated by cAMP; FFAR, free fatty acid receptor; GLP-1, glucagon-like peptide-1; GLP-1R, glucagon-like peptide-1 receptor; NDCs, nondigestible carbohydrates; PEPT1, peptide transporter 1; SGLT1, sodium-glucose cotransporter 1; PKA, protein kinase A; VTA/NAc, ventral tegmental area/nucleus accumbens.

By binding to GLP-1Rs expressed on pancreatic *β*-cells, hypothalamic neurons, and enteric neurons, GLP-1 coordinates metabolic processes involved in glucose homeostasis and energy balance [[34], [35], [36]]. In pancreatic islets, GLP-1 enhances glucose-dependent insulin secretion, suppresses glucagon release, and supports *β*-cell survival and function, partly through intraislet paracrine signaling [36,37]. In the central nervous system, GLP-1 reduces both homeostatic and hedonic appetite drive, while peripherally delaying gastric emptying and enhancing satiety [38]. Together, these actions improve glycemic control, reduce caloric intake, and support obesity management [39]. These integrated effects linking intestinal nutrient sensing with appetite regulation and pancreatic function are summarized in Figure 2.

### Anti-inflammatory and immunological effects of dietary fibers

Although GLP-1RAs modulate systemic inflammation and immunity through GLP-1R signaling, dietary fibers offer a complementary and microbiota-mediated approach that supports gut-immune homeostasis and strengthens intestinal barrier function. Dietary fibers, in particular soluble fibers, are fermented in the gut by commensal bacteria, producing SCFAs such as acetate, propionate, and butyrate [40]. Both SCFAs and their dietary fiber precursors are key modulators of intestinal immune homeostasis. SCFAs signal through G protein–coupled receptors (GPCRs), e.g., GPR41, GPR43, and GPR109A, expressed on epithelial cells, dendritic cells, Tregs, neutrophils, mast cells, and innate lymphoid cells, and they inhibit histone deacetylases, thereby promoting core anti-inflammatory transcriptional programs [41]. Functionally, these pathways calibrate mucosal immunity by expanding Tregs and dampening proinflammatory cytokine production [42]. In parallel, dietary fibers and their fermentation products directly fortify the epithelial barrier by enhancing tight-junction assembly, stimulating goblet-cell mucus secretion, regulating epithelial growth, and preserving the glycocalyx integrity; they may also act via pattern-recognition receptors on epithelial and innate immune cells to further modulate immunity [41]. Mechanistically informed interventions illustrate these principles. Inulin acts in a microbiota-dependent manner to induce IL-22 production by group 3 innate lymphoid cells (ILC3s); IL-22 then promotes enterocyte proliferation and antimicrobial peptide expression (e.g., Reg3γ), limits microbiota encroachment, and reduces low-grade inflammation, thereby protecting against diet-induced obesity and metabolic syndrome [43]. Complementarily, pairing *Bacteroides uniformis* with wheat-bran extract in obese mice raises cecal butyrate and restores intraepithelial lymphocytes and ILC3s, strengthening first-line mucosal defense; it also attenuates dysregulated IL-22 signaling and hepatic inflammation while lowering interferon (IFN)-γ [44]. Consistent with these findings, red-ginseng dietary fiber in obese mice increased SCFAs and mucosal effectors such as *β*-defensin 2 and mucin 2 (MUC2), improved intestinal motility, and reduced markers of inflammation, e.g., C-reactive protein (CRP), inducible nitric oxide synthase, myeloperoxidase, and proinflammatory cytokines. It also produced histological recovery, which together indicates restoration of intestinal immune and barrier function [45]. These data indicate that dietary fibers regulate the gut microbiota and promote SCFA production via microbial fermentation, with both direct and indirect enhancement of gut barrier integrity and mucosal immune tolerance. These benefits accrue gradually and vary by fiber type and dose. By contrast, as shown in Figure 4, GLP-1RAs exert broad modulation of both innate and adaptive immune responses through GLP-1R signaling and, in clinical studies, generally achieve larger reductions in systemic inflammatory markers (e.g., CRP). Both strategies confer immune advantages relevant to effective obesity management and are potentially complementary.

**FIGURE 3:**
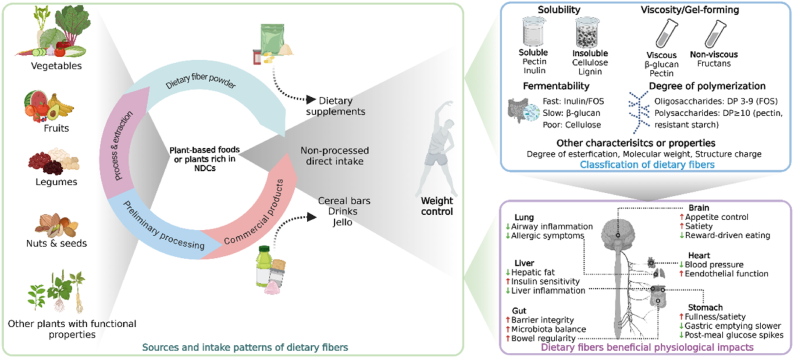
Dietary fibers: sources, classification, and systemic effects. This diagram provides an overview of the main classes of dietary fibers, soluble and insoluble, and their physiological actions. Soluble fermentable fibers such as inulin, *β*-glucans, and psyllium form viscous gels and are metabolized by colonic microbiota into SCFAs, which enhance GLP-1 release, improve lipid metabolism, and reduce postprandial glycemia. Insoluble fibers such as cellulose primarily contribute to fecal bulk and intestinal transit. Chronic fiber intake reshapes the gut microbiome, promotes epithelial integrity, and supports metabolic and immune resilience, providing a foundation for sustainable obesity management. DP, degree of polymerization; FOS, fructooligosaccharides; GLP-1, glucagon-like peptide-1; NDCs, nondigestible carbohydrates; SCFAs, short-chain fatty acids.

### Insights from preclinical studies

Dietary fiber encompasses NDCs and lignin that resist enzymatic digestion in the small intestine and reach the colon largely intact, where fermentable fractions are metabolized by the gut microbiota [46]. Fiber intake reduces subsequent energy intake and supports weight loss primarily by increasing satiation, lowering dietary energy density, and modulating gut–hormone signaling [47]. The magnitude of these effects depends on the physicochemical properties of each fermentable fiber, particularly viscosity and fermentability, which influence GLP-1 secretion [48].

Among highly fermentable fibers, greater viscosity has been associated with lower fasting GLP-1 concentrations. In a rat study comparing dietary fibers that differed in viscosity and fermentability, a nonviscous short-chain fructooligosaccharide (FOS) diet elicited the highest plasma GLP-1 concentrations, whereas a viscous *β*-glucan diet produced the lowest [49]. Nevertheless, chronic intake of fermentable fibers such as *β*-glucan can regulate the gut microbiota and increase colonic SCFA concentrations, which activate GPR43/FFAR2 and contribute to glucose lowering in obese mice [50]. Beyond effects on fermentation and the microbiota, dietary fiber supports L-cell secretory capacity and preserves intestinal morphology. In another rat study, 4 wk supplementation of the diet with 10% oligofructose doubled the density of GLP-1–positive L cells in the proximal colon and yielded ∼2-fold increases in colonic proglucagon mRNA and GLP-1 content [51]. Collectively, these findings show that fermentable dietary fibers can influence GLP-1 secretion through microbiota-dependent fermentation, SCFA production, and preservation of intestinal morphology and L-cell function. An overview of dietary fiber sources, classifications, and system effects in obesity management is summarized in Figure 3.

**FIGURE 4:**
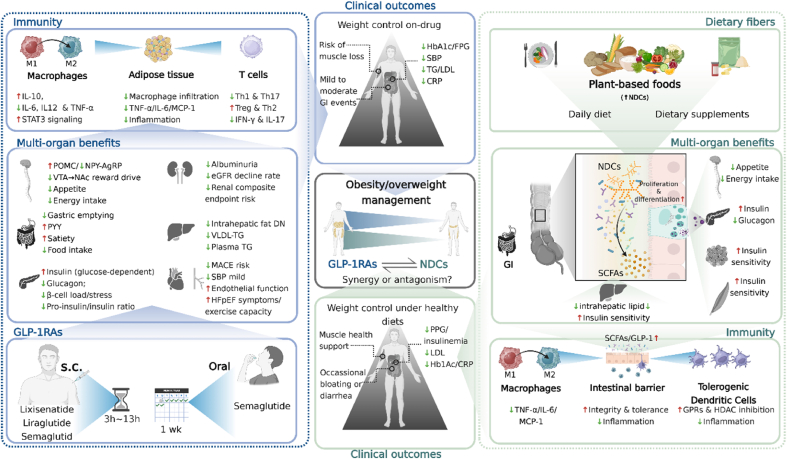
GLP-1RAs and dietary fibers: convergent and complementary mechanisms in weight management. This composite figure contrasts the principal mechanisms of GLP-1RAs and dietary fibers in metabolic regulation. GLP-1RAs activate systemic GLP-1 signaling to suppress appetite, enhance insulin secretion, and promote weight loss, whereas dietary fibers modulate the gut microbiota and stimulate endogenous GLP-1 production through fermentation-derived SCFAs. Both interventions attenuate inflammation, improve lipid and glucose metabolism, and support cardiovascular health. The overlap in pathways highlights their potential complementarity in combined obesity therapy. CRP, C-reactive protein; DN, diabetic nephropathy; eGFR, estimated glomerular filtration rate; FPG, fasting plasma glucose; GI, gastrointestinal; GLP-1, glucagon-like peptide-1; GLP-1RA, glucagon-like peptide-1 receptor agonist; GPR, G protein-coupled receptor; HbA1c, hemoglobin A1c; HDAC, histone deacetylase; HFpEF, heart failure with preserved ejection fraction; IFN-γ, interferon gamma; MACE, major adverse cardiovascular events; MCP-1, monocyte chemoattractant protein-1; NAc, nucleus accumbens; NDCs, nondigestible carbohydrates; NPY-AgRP, neuropeptide Y/agouti-related peptide; POMC, pro-opiomelanocortin; PPG, preproglucagon; PYY, peptide tyrosine tyrosine; SBP, systolic blood pressure; SCFAs, short-chain fatty acids; STAT3, signal transducer and activator of transcription 3; TG, triglycerides; Treg, regulatory T cell; VLDL, very-low-density lipoprotein; VTA, ventral tegmental area.

### Summary of clinical interventions with dietary fibers

Increasing dietary fiber intake is a nutritional strategy for managing obesity. Nevertheless, their effects on body weight are smaller than those achieved with GLP-1RA pharmacotherapy. Figure 3 summarizes the key mechanistic and clinical outcomes associated with dietary fibers in obesity treatment. In a meta-analysis of 62 trials, adding viscous fibers such as psyllium, glucomannan, and *β*-glucans to an ad libitum diet produced a mean additional weight loss of ∼0.3 kg relative to control [19]. Even when combined with energy restriction, fiber supplementation yields modest incremental loss; across 15 diet trials, the addition of fiber resulted in an extra ∼0.8 kg reduction compared with diet alone [52]. Some trials report stronger effects. For example, in an 8-wk hypocaloric diet study, individuals who received a mixed-fiber supplement containing glucomannan, inulin, and psyllium lost ∼4 to 5 kg. In contrast, those in the placebo group (diet alone) lost ∼3 kg. Likewise, meta-analyses in individuals with type 2 diabetes mellitus (T2DM) indicate that soluble fiber improves glycemic control while also reducing BMI [53]. Recent evidence indicates that GLP-1RAs produce smaller weight-loss effects in individuals with T2DM than in those without diabetes [54,55]. Accordingly, the additional reductions in body weight and improvements in glycemic control may be associated with soluble fiber and warrant emphasis as a complementary strategy in this population. Collectively, GLP-1RAs remain substantially more potent, typically inducing 10% to 15% weight loss in clinical populations. Even so, dietary fiber supplementation provides small but measurable benefits for BMI management and may serve as a supportive adjunct. Detailed efficacy summaries are presented in Table 1 [12,20,53,[56], [57], [58], [59], [60], [61], [62], [63], [64]].

**TABLE 1:** Clinical evidence on dietary fibers and GLP-1RAs in obesity/overweight

Intervention type	Model/population	Duration	Treatment	GLP-1 pathway involvement	Effects in obesity	Metabolism	Immunity	Safety and tolerability	Reference
Dietary fiber	Adults with overweight/obesity	8 wk, crossover	Resistant starch (40 g/d) vs. control starch	Not directly tested	Reduced body weight, fat mass, waist circumference, and visceral fat	Improved insulin sensitivity and glucose tolerance; increased ANGPTL4	Reduced TNF-α and IL-1β	NR	[56]
	Adults from an observational multiomics cohort	Observational	Habitual dietary fiber intake	Not directly tested	Not an obesity intervention	Higher fiber intake associated with lower CRP, especially with abundant *Prevotella copri*	Lower systemic inflammation	NR	[57]
	Adults with overweight/obesity undergoing supervised exercise	12 wk	Wheat-pasta vs. Mediterranean diet × low-fiber vs. high-fiber diet	Fasting and postprandial GLP-1 unchanged; PYY increased in one subgroup	No significant change in body weight or composition	Energy expenditure and insulin sensitivity unchanged	NR	NR	[58]
	Children with obesity	6 mo	Oligofructose-enriched inulin vs. maltodextrin vs. dietary fiber advice	NR	BMI *z*-score and body fat decreased across all groups, with no between-group difference	No significant change in fasting glucose, lipids, or ALT	IL-1β and TNF-α decreased over time	Dropout 6%; adverse events NR	[59]
	Healthy adults, randomized crossover acute meal study	Acute postprandial test	Resistant-polysaccharide products vs. controls	Increased GLP-1, particularly with capsule and pectin conditions	Increased satiety and reduced hunger	Reduced postprandial glucose with powder; insulin increased with powder	NR	Mild flatulence only	[60]
	Adults with T2DM, meta-analysis of RCTs	Varied	Soluble fiber supplementation	Not consistently reported across trials	Modest BMI reduction	Improved HbA1c, fasting glucose, postprandial glucose, fasting insulin, and HOMA-IR	NR	NR	[53]
GLP-1RA	Adults with overweight/obesity, no diabetes	68 wk	Semaglutide 2.4 mg s.c. once weekly + lifestyle	Direct GLP-1R activation by agonist	Marked weight loss	Improved BP, lipids, and HbA1c	Reduced hs-CRP	GI adverse events common; GI-related discontinuation higher than placebo	[20]
	Adults with overweight/obesity, with or without prediabetes	104 wk	Semaglutide 2.4 mg s.c. once weekly + behavioral intervention		Sustained weight loss and reduced waist circumference	Improved SBP, glycemic control, insulin, and lipid profile	Reduced hs-CRP	GI adverse events common; discontinuation slightly higher than placebo	[61]
	Adults with overweight/obesity after semaglutide run-in	20-wk run-in + 48-wk randomized phase	Continued semaglutide 2.4 mg weekly vs. placebo + lifestyle		Continued treatment caused further weight loss; withdrawal led to regain	Metabolic benefits were better maintained with continued treatment	NR	GI disorders more common with semaglutide; serious adverse events similar	[12]
	Woman with overweight/obesity	52 wk	Exenatide s.c. twice-daily vs. diet/placebo		Modest weight loss	Reduced TG, total cholesterol, and LDL-C	No treatment-related CRP change reported	Nausea more common; serious adverse events similar	[62]
	Adults with obesity, no diabetes	6 mo	Once-weekly semaglutide titrated to 1 mg		BMI decreased; some participants achieved >5% weight loss	HbA1c decreased in some participants	Enhanced NK-cell activity and cytotoxicity	NR	[63]
	Adults with T2DM	30–56 wk	Semaglutide 0.5 or 1.0 mg once weekly vs. comparators		Reduced body weight	Improved HOMA-IR, largely mediated by weight loss	NR	GI adverse events common, generally mild-to-moderate	[64]

This table summarizes clinical evidence from intervention, observational, and meta-analysis studies of dietary fibers and GLP-1RAs in obesity, overweight, or related metabolic disease populations.

Abbreviations: ALT, alanine aminotransferase; ANGPTL4, angiopoietin-like 4; BP, blood pressure; CRP, C-reactive protein; GI, gastrointestinal; GLP-1, glucagon-like peptide-1; GLP-1RA, glucagon-like peptide-1 receptor agonist; HbA1c, glycated hemoglobin; hs-CRP, high-sensitivity C-reactive protein; NK, natural killer; NR, not reported; PYY, peptide tyrosine tyrosine; RCT, randomized controlled trial; SBP, systolic blood pressure; s.c., subcutaneous; T2DM, type 2 diabetes mellitus; TG, triglycerides.

Dietary fiber intake is associated with improvements in metabolic health, and the efficacy of the effect depends on dose, duration, and fiber chemistry. In a meta-analysis of patients with diabetes, consuming ∼8 g/d of soluble fiber led to a mean reduction in (hemoglobin A1c) HbA1c of ∼0.6 percentage points compared with controls. Although clinically meaningful, this reduction is roughly half of what is typically achieved with GLP-1RAs [53]. The benefits brought by dietary fiber intake need to be maintained through long-term consumption. For glycemic control, a 2-y trial showed that consuming >14 g/d of insoluble oat-hull fiber produced a clear, dose-dependent effect. Participants experienced a reduction of ∼0.8 mmol/L in 2-h postprandial glucose concentrations. At the same time, both fasting and postprandial insulin sensitivity improved [58]. By contrast, in an 8-wk energy-restricted trial, adding mixed soluble fibers, including glucomannan, inulin, psyllium, and *β*-glucans, did not lower fasting glucose beyond diet alone [65]. With respect to lipids, the same mixed-fiber intervention produced greater reductions in total and LDL cholesterol than placebo after 4 wk [65]. However, these between-group differences diminished by week 8, suggesting the lipid-lowering effect attenuated over time. Weight loss from any cause typically lowers blood pressure [66]. By assisting with weight management, dietary fibers can produce modest reductions in blood pressure, though the magnitude of reduction is generally more modest than that reported with GLP-1RAs. A meta-analysis of 22 randomized controlled trials found that adding ∼9 g/d of viscous soluble fibers for roughly 7 wk significantly reduced systolic blood pressure by ∼1.6 mm Hg, and diastolic blood pressure fell by ∼0.4 mm Hg. Among the fibers tested, psyllium showed the strongest effect [67]. Although small at the individual concentration, such reductions can translate into meaningful population-level decreases in cardiovascular risk.

Overall, fiber supplements consistently improve key metabolic markers, including glycemic control, lipid profiles, and blood pressure. Yet, the effect sizes are generally smaller than those seen with GLP-1RAs. Notably, fiber benefits accrue gradually and are sustainable with long-term intake; beyond metabolic endpoints, fibers support a balanced gut microbiota and strengthen intestinal barrier function [68,69]. These properties make dietary fibers a rational foundation for combination strategies with GLP-1RAs, potentially enhancing therapeutic outcomes and supporting weight maintenance as pharmacotherapy is tapered.

## Role of GLP1-RAs in Obesity and Weight Management

### Mechanisms of the prolonged action of GLP-1RAs

GLP-1RAs are synthetic peptides engineered to activate the GLP-1R with more optimal pharmacokinetic and dynamic properties compared with native GLP-1. They are broadly classified as analogs of the human GLP-1 sequence (e.g., liraglutide, semaglutide) or as exendin-4–based molecules (e.g., exenatide, lixisenatide). Native GLP-1 is quickly inactivated by DPP-4 and cleared renally, resulting in a plasma half-life of only 2 to 3 min. In contrast, GLP-1RAs hold several targeted modifications, such as specific amino acid substitutions reducing DPP-4 susceptibility, fatty acid acylation to promote albumin binding, and fusion to large carrier proteins, e.g., albumin, increasing the molecular size (from ∼3 to >59 kDa) to slow glomerular filtration, thereby prolonging systemic exposure. These engineered features extend half-lives to hours and even days, enabling once- or twice-daily and even once-weekly dosing [70,71]. At the same time, their preserved receptor selectivity minimizes off-target signaling. This selectivity supports cardiovascular benefits and weight-loss effects, which increasingly position GLP-1RAs as multisystem therapeutics for obesity [72].

### Anti-inflammatory and immunological effects of GLP-1RAs

Even though GLP-1RAs are commonly used in adults with T2DM and/or obesity for glycemic control and weight loss, broader evidence further supports that GLP-1RAs exert anti-inflammatory and immunological actions across multiple tissues and disease settings, highlighting that their benefits extend beyond metabolic control alone [73]. As GLP-1 mimetics, they engage GLP-1Rs expressed on diverse immune cells, including T lymphocytes, thereby modulating immune activity and dampening inflammation [74]. In diabetic mice with obesity, pharmacologic augmentation of GLP-1 signaling markedly reduced adipose tissue inflammation by limiting proinflammatory macrophage infiltration, lowering TNF-α, IL-6, and monocyte chemoattractant protein-1 (MCP-1) expression, and attenuating nuclear factor kappa B (NF-κB) and c-Jun N-terminal kinase (JNK) activation in adipocytes. GLP-1R agonism also decreased M1 macrophage markers while sparing M2 markers, indicating a shift away from proinflammatory macrophage polarization [75]. Across cardiovascular and neuroinflammatory models, GLP-1RAs similarly bias tissue macrophages and microglia toward anti-inflammatory states and suppress inflammasome-mediated cytokine release [76,77]. Consistent with these findings, activation of the GLP-1Rs attenuates innate inflammatory signaling in other settings. Treatment with GLP-1RAs reduces Toll-like receptor–induced cytokine release, limiting production of TNF-α, IL-1*β*, and IL-6 while increasing the anti-inflammatory cytokine IL-10 [[78], [79], [80]]. In the adaptive immune compartment, agents such as liraglutide dampen effector T-cell activity and enhance Treg responses [81]. *In vitro*, liraglutide suppresses Th1 (CD4^+^IFN-γ^+^) and Th17 (CD4^+^IL-17A^+^) populations and promotes anti-inflammatory phenotypes, increasing Th2 (CD4^+^IL-4^+^) and Treg (CD4^+^CD25^+^/FoxP3^+^) cells [82]. Similarly, exendin-4 corrects Th17/Treg imbalance implicated in autoimmunity and inflammation by promoting Treg expansion and restraining Th17 infiltration [82]. GLP-1R expression is highest on T cells under Treg polarizing conditions, and GLP-1R–positive T cells are enriched for FoxP3 and CD25 [83]. Recent mechanistic work further showed that GLP-1R acts as a T cell–negative costimulatory molecule, providing direct support for the ability of GLP-1 signaling to restrain T-cell activation and immune-mediated injury [84]. In addition, in cardiometabolic patients with T2DM, semaglutide lowered CRP by 42.0% compared with 12.8% with placebo, underscoring its anti-inflammatory benefits beyond glycemic control [85]. The broader translational relevance of these anti-inflammatory effects is increasingly being recognized beyond metabolic disease, including neurodegenerative settings in which modulation of neuroinflammation may contribute to therapeutic benefit [86]. Figure 4 illustrates how GLP-1RAs modulate inflammatory pathways in macrophages, adipose tissue, and T cells, and how these immune effects contribute to obesity management.

### Insights from preclinical studies

Mechanistic studies further support the clinical efficacy of GLP 1RAs. In preclinical mouse models of both diet-induced and genetic obesity, treatment with liraglutide was shown to modulate gut microbiota composition, reduce hepatic steatosis, and promote sustained weight loss [87]. However, whether these microbiota changes reflect direct drug effects or arise secondarily from altered food intake, GI transit, and weight reduction remains uncertain. Semaglutide is now one of the most widely used GLP-1RAs, combining potent glucose lowering, clinically meaningful weight loss, cardiovascular benefits, and a 1-wk half-life and pharmacodynamic effects. In high-fat diet–induced obese mice, semaglutide (100 μg/kg) reduced body weight and fat mass and improved fasting glucose and insulin. Transferring fecal microbiota from semaglutide-treated donors to antibiotic-depleted pseudo-germ-free obese mice partially reproduced metabolic benefits, such as lower fasting insulin and improved insulin resistance and lipids, implicating microbiome-mediated mechanisms [88]. Their effects on the microbiome may reflect both potential direct drug actions and, importantly, changes secondary to altered dietary profiles and reduced food intake. In addition, in a model of sarcopenic obesity, semaglutide improved muscle quality by reducing intramuscular fat, enhancing mitochondrial density, and increasing type I muscle fiber area, thereby addressing concerns regarding potential adverse impacts on lean mass during weight loss [89]. These agents represent comprehensive therapeutic strategies capable of effectively reducing adiposity, enhancing muscle quality, and improving metabolic health. Key preclinical findings are summarized in Table 2 [44,82,84,[90], [91], [92], [93], [94]].

**TABLE 2:** Preclinical evidence on dietary fibers and GLP-1RAs in obesity/overweight

Intervention type	Model	Duration	Treatment	GLP-1 pathway involvement	Effects in obesity	Metabolism	Immunity	Reference
Dietary fiber	T2DM mice (HSHFD + STZ), with FMT validation	8 wk	*Achyranthes bidentata* polysaccharide	Increased colonic GLP-1, GLP-1R, cAMP, PKA, CREB, and GPR43; FMT reproduced effects	Attenuated diabetes-associated weight loss	Improved fasting glucose, OGTT, insulin, lipid profile, and SCFA production	Improved gut barrier and microbiota profile	[90]
	Mice with HFHSD-induced obesity	17 wk	*B. uniformis* CECT 7771 ± wheat-bran extract	GLP-1 normalized; PYY increased	Reduced weight gain and epididymal fat mass	Improved glucose tolerance, plasma lipids, cecal butyrate, and bile acid profile	Improved colonic barrier and mucosal immune markers	[44]
	HFD-fed mice	12 wk	Tremella polysaccharide	Increased serum GLP-1 and GLP-2	Reduced body weight gain, fat mass, and adipocyte size	Improved fasting glucose, glucose tolerance, serum lipids, and hepatic steatosis	Reduced LPS, TNF-α, and IL-6	[91]
	C57BL/6J mice, including receptor knockout models	21 and 112 d	Fiber-free diet vs. chow	Fiber deprivation reduced GLP-1, GLP-2, and PYY	Body weight decreased during short-term fiber deprivation	Intestinal permeability increased after long-term fiber deprivation	Gut morphology deteriorated under fiber deprivation	[92]
GLP-1RA	Diet-induced obese male mice	7 d clamp + 28 d dosing	Cotadutide or g1437 vs. liraglutide vs. vehicle	Direct GLP-1R activation by agonist	Dual agonists reduced body weight more than liraglutide	Improved insulin sensitivity, glucose disposal, BAT uptake, energy expenditure, and liver lipid content	NR	[93]
	Diabetic/obese mouse models with implant-related infection	19 and 28 d	Semaglutide 120 μg/kg s.c. every 3 d		Significant and sustained body weight reduction	Improved glucose tolerance	Enhanced neutrophil function and reduced infection rates	[94]
	db/db mice	8 wk	Exenatide 200 μg/kg		Reduced early weight gain, though not sustained at 8 wk	Improved fasting glucose, insulin resistance, and glucose tolerance	Reduced Th17/IL-17 responses; increased Treg-related signals	[82]
	High fat diet obese C57BL/6J mice	10 wk	Semaglutide 100 μg/kg every other day		Reduced body weight, fat mass, food intake, and hepatic lipid accumulation; rebound after withdrawal	Improved glucose homeostasis, insulin resistance, lipid profile, and lipid oxidation-related genes	Altered gut microbiota composition	[88]

This table summarizes preclinical studies of dietary fibers and GLP-1RAs in obesity, overweight, or related metabolic disease models.

Abbreviations: BAT, brown adipose tissue; *B. uniformis*, *Bacteroides uniformis*; CREB, cAMP response element-binding protein; FMT, fecal microbiota transplantation; GLP-1, glucagon-like peptide-1; GLP-1RA, glucagon-like peptide-1 receptor agonist; GLP-2, glucagon-like peptide-2; GPR43, G protein–coupled receptor 43; HFD, high-fat diet; HFHSD, high-fat high-sucrose diet; HSHFD, high-sugar high-fat diet; NR, not reported; PKA, protein kinase A; PYY, peptide tyrosine tyrosine; OGTT, oral glucose tolerance test; s.c., subcutaneous; SCFA, short-chain fatty acid; STZ, streptozotocin.

### Summary of key findings of clinical interventions with GLP-1RAs

GLP-1RAs are among the most effective pharmacotherapies for weight management and also achieve meaningful improvements in both glycemic control and body weight reduction in individuals with T2DM [95]. Among approved agents, the intermediate-acting lixisenatide (half-life of 2–4 h and pharmacodynamic effects lasting ∼6–12 h postdose) shows modest but clinically measurable weight effects. A recent meta-analysis of 26 intervention arms reported a mean reduction of ∼0.97 kg, with larger losses in shorter trials (<24 wk), at lower daily doses (<19 μg), and in older patients (≥60 y) [96]. Meanwhile, real-world data from 792 United Kingdom patients showed that the longer-acting GLP-1RA therapeutic liraglutide (half-life of 11–15 h and pharmacodynamic effects lasting ∼24 h postdose) achieved superior glycemic control and larger BMI reductions than the shorter-acting GLP-1RA lixisenatide, underscoring the greater effectiveness of longer-acting GLP-1RAs in routine practice [97]. The weight-loss effects of GLP-1RAs also vary among different populations. Among women with overweight or obesity treated with twice-daily immediate-release exenatide (half-life of ∼2.4 h and pharmacodynamic effects lasting ∼4–6 h postdose), weight-loss responses were highly heterogeneous. By 12 wk, 56% achieved ≥5% weight loss (high responders) and 23% achieved ≥10% loss (super responders), whereas the remainder did not reach these thresholds, underscoring the interindividual variability in treatment response [62]. Semaglutide has further strengthened the clinical position of this drug class. In the semaglutide treatment effect in people with obesity trial, once-weekly semaglutide 2.4 mg combined with lifestyle intervention resulted in a mean body weight reduction of 14.9% at 68 wk, compared with 2.4% in the placebo group, underscoring the substantial efficacy of GLP 1RAs in the treatment of obesity [20].

Overall, converging evidence from clinical trials and mechanistic studies provides robust support for the efficacy of GLP-1RAs across agents ranging from short-acting exenatide and intermediate-acting lixisenatide to long-acting liraglutide and semaglutide. Besides, emerging coagonist approaches, such as CagriSema (combination of cagrilintide and semaglutide), may yield even greater weight loss and broader metabolic benefits [98]. More recent real-world evidence also suggests that combination strategies may broaden implementation in the treatment of T2DM. Specifically, dapagliflozin plus oral semaglutide reduced HbA1c by 1.2% after 6 mo, compared with 0.5% with dapagliflozin alone, supporting the potential of combination therapy to improve glycemic control and even contribute to pharmacological remission in selected patients [99]. Key clinical outcomes are summarized in Table 1. Clinical and mechanistic benefits of GLP-1RAs are summarized in Figure 4. Together, these effects support GLP-1RAs as effective therapies in obesity management.

## Practical Considerations for Dietary Fibers and GLP-1RAs

### Tolerability and adverse effects

Obesity treatment often requires long-term therapy, so tolerability is crucial for maintaining adherence and minimizing discontinuation. Compared with GLP-1RAs, dietary fibers as food components are rarely associated with severe adverse events. Mild GI discomfort can occur, particularly when intake is increased too rapidly. However, gradual introduction generally yields excellent tolerability and supports safe, long-term intake [100]. Fermentable fibers, e.g., inulin, are readily metabolized by colonic bacteria with consequent gas production and therefore more often cause bloating and flatus [101]. By contrast, psyllium is a gel-forming, soluble, minimally fermentable fiber that typically produces less gas than inulin in patients with irritable bowel syndrome [102]. This pattern suggests that slowly fermentable or nonfermentable fibers may possess better tolerability than rapidly fermentable prebiotics such as FOS and raffinose [102,103]. Pragmatic strategies can minimize GI effects during fiber supplementation for obesity management. Increasing intake gradually over several weeks, titrating toward ∼20 to 30 g/d, may limit bloating and flatulence, although benefits may take weeks to manifest [104]. In the 8-wk trial of various fiber supplements, all fiber groups reported some GI symptoms such as mild bloating, but overall, no serious adverse events were noted, and dropout rates were low and similar to placebo [65]. Overall, the adverse effects of dietary fibers are predominantly GI and typically mild. Selecting appropriate fiber types, adjusting the dose carefully, and introducing them gradually can substantially mitigate symptoms. Once adaptation occurs, most individuals tolerate fiber well [105,106]. This makes dietary fiber supplementation a safe, practical, and sustainable long-term strategy to support obesity management and overall metabolic health.

For obesity management, GLP-1RA therapy and dietary fiber supplements have distinct adverse event profiles, although GI symptoms are common to both. For GLP-1RAs, GI effects are typically dose-dependent and most prominent during initiation and dose escalation. The most frequently reported events are nausea, vomiting, diarrhea, and constipation [107]. In a 68-wk trial of once-weekly semaglutide for obesity, GI adverse events were more frequent in the semaglutide group than in the placebo group. Nausea was reported in 44.2% compared with 17.4% of participants, vomiting in 24.8% compared with 6.6%, diarrhea in 31.5% compared with 15.9%, and constipation in 23.4% compared with 9.5%. These events were generally transient and of mild-to-moderate severity but resulted in treatment discontinuation in 4.5% of semaglutide-treated participants compared with 0.8% in the placebo group [20]. Most events were mild-to-moderate and tended to improve over time or with dose adjustment. Additional adverse effects such as dizziness, fatigue, headache, mild hypoglycemia, and injection site reactions are generally manageable [108]. Although GI symptoms may be mild in the general population, special consideration is warranted in older or frail individuals, in whom nausea and dehydration may exacerbate frailty or precipitate clinical deterioration [109]. Overall, GLP-1RAs are considered safe for most patients, with GI intolerance representing the principal trade-off for their efficacy. Careful, gradual dose titration improves tolerability [54].

### Durability and other trade-offs

Evidence indicates that after an initial phase of rapid weight loss, GLP-1RAs can help sustain weight reduction. In a 2-y clinical trial with continuous semaglutide treatment, participants maintained a mean weight loss of ∼15% at 104 wk, compared with ∼2.6% in the placebo group [61]. Cessation of therapy often results in regaining a substantial portion of the lost weight within months, reflecting the chronic, relapsing nature of obesity. These dynamics are illustrated by a study in which participants received semaglutide for 20 wk and lost ∼10.6% of body weight, after which they were randomly assigned to continue semaglutide or switch to placebo. Those who continued semaglutide lost an additional 7.9% over the next 48 wk, whereas those who stopped regained ∼6.9% [12]. This dependency on ongoing use underscores a limitation of pharmacotherapy. Its benefits rely on long-term adherence, which may be constrained by cost, adverse effects, or patient preference. Sustainability remains a concern because long-term adherence and access can be limited by high medication costs and the need for frequent injections, which may reduce persistence outside trial settings [110]. In a large United States cohort of adults with overweight or obesity who initiated liraglutide, semaglutide, or tirzepatide, 53.6% discontinued treatment within 1 y [111]. Patients who achieved greater weight loss were less likely to discontinue, whereas substantial GI adverse effects and socioeconomic barriers were associated with higher discontinuation [111,112]. These findings indicate that the long-term success of GLP-1RAs strongly depends on continued use. Once treatment stops, benefits tend to diminish rapidly. Another important trade-off is that GLP-1RA–induced weight loss may include loss of lean body mass, so in older, frail, or otherwise high-risk patients at risk of sarcopenia, preservation of muscle mass and function should be considered an explicit treatment goal during therapy [113].

In contrast to pharmacotherapy, dietary fiber supplements are intended as a practical, long-term adjunct to help individuals who do not consistently achieve recommended fiber intakes from food. When used to close this intake gap, long-term supplementation is generally safe and does not confer risk of systemic drug adverse effects. For example, daily intake of ∼10 g of psyllium before meals forms a minimally fermented, viscous gel that slows nutrient absorption, improves insulin sensitivity, and favorably modifies lipid profiles. Over 5 mo, this intervention reduced body weight by 2.1 kg, BMI by 0.8 kg/m^2^, and waist circumference by 2.2 cm in adults with overweight or obesity (*n* = 354) [114]. In practice, fiber works best as part of a healthy diet and lifestyle program. It can modestly increase satiety, facilitate caloric control, and improve metabolic health by lowering cholesterol and stabilizing postprandial glycemia [115,116]. Because fiber is inexpensive, widely available, and associated with minimal adverse effects, patients are often more willing to continue its use [21,117]. Fiber, therefore, should be framed less as a stand-alone solution and more as a supportive, scalable component of long-term weight management, particularly for weight maintenance and overall metabolic health rather than large, rapid weight loss.

## Interactions between Dietary Fibers and GLP-1RAs

### Effects on gut microbiota and fermentation

Changes in the gut microbiota and fermentation during GLP-1RA therapy are likely driven predominantly by indirect effects, particularly reduced energy intake and accompanying shifts in dietary composition, with any direct drug effects on microbes remaining uncertain [118,119]. In obese mice, chronic liraglutide treatment induced distinct alterations in the gut microbiota, characterized by decreased abundances of certain Firmicutes taxa, including *Lachnospiraceae*, many of which are fiber fermenters, and a concomitant increase in *Akkermansia* [118]. In humans, evidence is heterogeneous; a placebo-controlled randomized trial in adults with T2DM found that 12 wk of liraglutide did not significantly change fecal α or *β* diversity relative to placebo [120]. Another characteristic effect of GLP-1RAs is the slowing of GI motility and transit, which mainly delays the timing of drugs or nutrient delivery to the intestine [121]. However, across clinical studies, it remains difficult to separate microbiota changes driven by altered GI transit from those attributable to weight loss, dietary shifts, or concomitant medications, which is a key limitation when interpreting GLP-1RA effects on microbiota [122]. Changes in transit time may indirectly modulate the gut microbiota by altering the steady supply of fermentable substrates e.g., slower colonic transit can limit carbohydrate availability and shift fermentation toward protein use [123]. Consistent with this mechanism, a systematic review reported that GLP-1RAs can enrich metabolically favorable taxa such as *Akkermansia* and *Bacteroides*, while in some settings reducing overall microbial diversity [122]. Notably, this liraglutide-induced *Akkermansia* enrichment mirrors that of inulin and butyrate in T2DM [124]. Mechanistically, delayed gastric emptying, together with reduced energy intake, lowers the quantity and slows the delivery of nutrients reaching the colon. This can regulate fermentation patterns, influence SCFA production from dietary fiber, and alter both the composition and function of the gut microbiota [125]. Taken together, current evidence suggests that GLP-1RA therapy may modify the colonic environment and fermentation context in which dietary fibers act, but direct causal effects of GLP-1RAs on gut microbiota in humans remain insufficiently established.

### GI adverse events

GI adverse effects are common with GLP-1RA, with clinical trials reporting incidences of 40% to 70% and some protocols reporting ≤85% [11]. Dietary fiber can produce a similar symptom profile, but it is usually milder and depends strongly on the fiber type and dose. In mice, high doses of soluble fibers such as FOS or xylooligosaccharides have produced flatulence, diarrhea, and microbiota dysbiosis [126]. These observations raise the hypothesis that GLP-1RAs and dietary fiber could have additive GI adverse effects, particularly at higher fiber intakes or in sensitive individuals, although this has not been established in controlled clinical trials. To reduce this risk, clinical guidance for patients using GLP-1RAs recommends careful fiber management. At dinner, patients are encouraged to choose low-fiber vegetables, avoid whole grains and high-fiber legumes during dose escalation, maintain adequate hydration, pair meals with moderate portions of complex carbohydrates and low-fat proteins, and increase the drug dose gradually to minimize GI discomfort [127]. Excessive fiber intake, or the use of fibers with specific fermentation properties, may exacerbate GI symptoms associated with GLP-1RA therapy. Although human evidence remains limited, interactions among drugs, diet, and the microbiota warrant further study because they may help explain mixed outcomes of fiber interventions in patients receiving incretin therapies and underscore the importance of comanaging diet and medication to preserve combined efficacy [128].

### Receptor occupancy and signaling interactions

GLP-1Rs are widely expressed across multiple cell types, as well as on enteroendocrine L cells and enteric neurons. Because long-acting GLP-1RAs, for example, exenatide or liraglutide, are designed for sustained exposure, high circulating concentrations can occupy and activate a large fraction of available GLP-1Rs over time [129]. Sustained or repeated GLP-1 exposure can reduce receptor responsiveness, leading to GLP-1R desensitization. This is often attributed to phosphorylation and *β*-arrestin–mediated internalization, which decreases surface receptor availability and cyclic AMP signaling [130]. For example, in *β*-cells, GLP-1 rapidly drives GLP-1R clustering and internalization into Rab5-positive endosomes with *β*-arrestin-2 engagement and increased lysosomal targeting. Prolonged GLP-1 exposure then diminishes GLP-1R–dependent cAMP signaling [131]. Although prolonged exposure to GLP-1 or its agonists can produce homologous desensitization in vitro, short-term in vivo treatment in mice (twice-daily intraperitoneal exendin-4 for 7 d) maintained glucose-lowering efficacy without clear tachyphylaxis [132]. Moreover, twice-daily exendin-4 continued to reduce HbA1c and postprandial glycemic excursions through 30 wk in clinical study participants [133]. Thus, in vitro desensitization does not necessarily translate into clinical resistance in vivo because receptor recycling and heterogeneous GLP-1R expression across tissues may preserve efficacy, although some effects, particularly those mediated by gastric and neuronal pathways, can attenuate over time [132,134].

**FIGURE 5:**
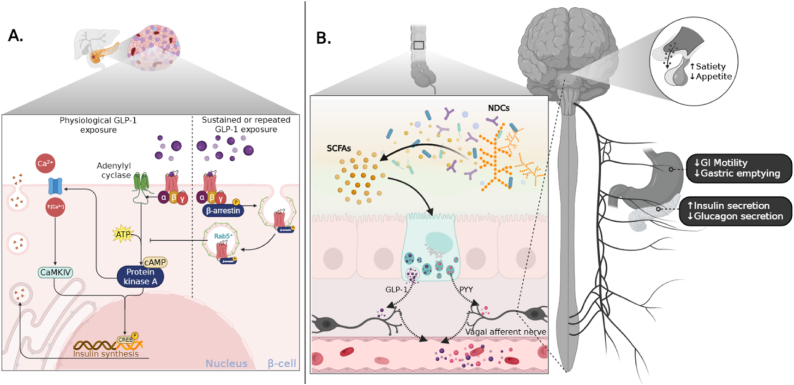
Receptor desensitization in *β* cells and compensatory nutrient-derived signaling during GLP-1R activation. Prolonged pharmacological GLP-1R stimulation can trigger receptor internalization and *β*-arrestin–mediated desensitization, attenuating cyclic AMP signaling in target cells. Despite this, tissue-specific receptor recycling maintains therapeutic efficacy. Concurrently, dietary fibers generate non-GLP-1 satiety signals, including peptide YY and SCFA-mediated vagal activation, which operate independently of GLP-1 receptor occupancy. This figure summarizes how endogenous nutrient-driven pathways may complement pharmacological GLP-1R activation and sustain appetite regulation during long-term therapy. CaMKIV, calcium/calmodulin-dependent protein kinase IV; GI, gastrointestinal; GLP-1, glucagon-like peptide-1; GLP-1R, glucagon-like peptide-1 receptor; NDCs, nondigestible carbohydrates; SCFAs, short-chain fatty acids.

Collectively, evidence for GLP-1RA–induced desensitization is mixed between in vitro and in vivo settings, and there is little indication to date of clinically meaningful GLP-1R desensitization during long-term GLP-1RA therapy, given the substantial and durable effects on weight, metabolism, and related outcomes. Therefore, if pharmacological GLP-1RAs achieve high GLP-1R occupancy, additional endogenous GLP-1 released after dietary fiber fermentation may contribute less to further receptor activation, although direct in vivo evidence for this hypothesis remains limited. Nevertheless, dietary fibers can stimulate additional GI satiety pathways, for example, peptide YY, which may enhance satiety in parallel with GLP-1 signaling [135]. These parallel and potentially overlapping mechanisms, including *β*-cell signaling downstream of GLP-1R activation, vagal and central appetite regulation, and NDCs-induced gut–hormone release, are illustrated in Figure 5. Therefore, concurrent intake of dietary fibers during GLP-1RA treatment may still enhance satiety through these parallel pathways. Currently, it remains uncertain whether endogenous GLP-1 acts synergistically with GLP-1RAs or competes for the same receptors. If competition occurs, endogenous GLP-1 would likely be disadvantaged because of its brief plasma half-life relative to long-acting GLP-1RAs. Direct *in vivo* evidence for receptor saturation is limited; however, this potential interaction highlights the importance of timing and dosing. Fiber-induced increases in endogenous GLP-1 may be more influential during troughs in GLP-1RA concentrations.

## Future Perspectives: Combined and Sequential Strategies

### Combined therapy

The combined and sequential pathways discussed below are intended as conceptual models to organize current evidence and highlight testable clinical hypotheses. They should not be interpreted as validated treatment strategies because direct randomized evidence comparing fiber first, GLP-1RA-first, maintenance, or step-down sequencing approaches remains limited.

To date, no randomized clinical trial has directly evaluated the additive or synergistic effects of dietary fiber supplementation when combined with GLP-1RA therapy compared with GLP-1RA monotherapy for weight loss. This represents an important evidence gap because GLP-1RAs commonly reduce meal size and overall caloric intake, potentially leading to suboptimal dietary fiber consumption and attenuating the metabolic and gut health benefits associated with adequate fiber intake [136]. In a recent cross-sectional study, adults using GLP-1RAs consumed a mean of 14.5 g of fiber per day, well below guideline targets [137]. Clinical guidance generally recommends pairing GLP-1RA therapy with a balanced dietary pattern that meets daily fiber targets (21–25 g/d for women, 30–38 g/d for men), alongside regular physical activity. This combination supports weight loss, helps preserve lean mass, and maintains adequate micronutrient intake during treatment [138]. Although dietary fiber is not a stand-alone treatment for sarcopenia, higher fiber intake has been associated with greater skeletal muscle mass and strength in adults aged ≥40 y, supporting fiber-rich dietary patterns as part of a broader muscle-preserving strategy during GLP-1RA therapy [139]. In fact, expert recommendations for managing GLP-1RA–related GI adverse effects frequently include psyllium or other soluble dietary fibers, which can relieve constipation and support digestive health, especially when accompanied by adequate hydration [11]. In practice, fiber should be encouraged rather than avoided during GLP-1RA therapy.

From a weight-management perspective, combining a fiber-rich dietary pattern (for example, legumes, vegetables, whole fruits, and whole grains instead of refined, energy-dense foods) with GLP-1RA therapy could plausibly provide additional appetite control and further reduce energy intake, although this remains to be tested directly in randomized trials. Although the medication is the principal driver of weight loss, coupling it with an appropriate dietary pattern offers a more holistic and sustainable approach [140]. Some attention to timing and tolerance is warranted when introducing fiber during GLP-1RA therapy. To minimize discomfort such as bloating or excess gas, patients should increase fiber gradually and allow time for GI adaptation [141]. Because GLP-1RAs slow gastric emptying, adding fiber can intensify early satiety or transient GI symptoms. If nausea or discomfort occurs, adjusting fiber type and dose to match individual tolerance is advisable; gradual up-titration and avoiding abrupt increases in bulk can improve comfort [127].

### Sequential strategies: benefits and concerns

**FIGURE 6:**
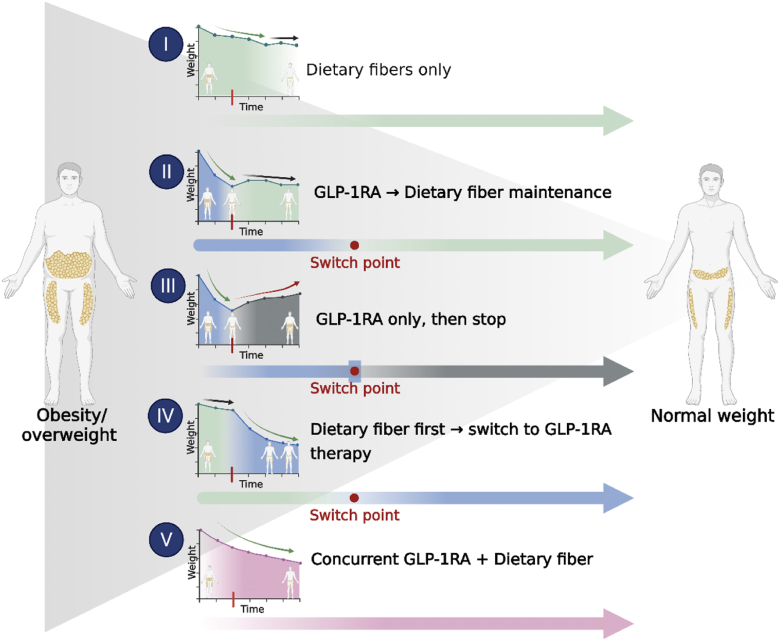
Sequential and combined strategies integrating GLP-1RAs with dietary fiber interventions. The figure outlines practical therapeutic pathways for integrating dietary fiber with GLP-1 receptor–agonist therapy. In a fiber-first strategy, increased intake of fermentable fibers promotes modest weight loss, improves microbiota composition, and enhances endogenous GLP-1 release, potentially delaying the need for pharmacotherapy. In a GLP-1RA–first or combined approach, fibers support GI comfort, mitigate constipation, and stabilize metabolic improvements during maintenance or drug tapering. Together, these strategies provide a continuum of care that aligns pharmacological potency with nutritional sustainability for long-term obesity management. GLP-1, glucagon-like peptide-1; GLP-1RA, glucagon-like peptide-1 receptor agonist.

A lifestyle-first approach is prudent for patients with obesity who do not have urgent indications for pharmacotherapy because it provides broad cardiovascular and metabolic benefits [142,143]. Consistent with this strategy, a fiber-first sequence begins with a high fiber, calorie-restricted diet or extra fiber supplementation plus exercise, reserving antiobesity medication for inadequate response. This aligns with guidance that positions lifestyle modification as the foundation of obesity care and states that GLP-1RA therapy should be used in conjunction with diet and physical activity [144]. A practical overview of candidate care pathways (fiber first, GLP-1RA-first, switch or maintenance, and concurrent) is provided in Table 3, and candidate sequencing strategies are illustrated in Figure 6. Dietary fiber interventions, whether by increasing vegetables and whole grains in the habitual diet or extra fiber supplements (e.g., psyllium or other fiber supplement capsules), can deliver meaningful health benefits and moderate weight loss even as stand-alone measures [145,146]. A fiber-first strategy may suit patients who prefer to avoid medications initially, those with milder obesity or individuals with overweight, and those with contraindications to GLP-1RAs, for example, a history of medullary thyroid carcinoma or severe GI disorders [147]. A potential limitation is that diet-induced weight loss is often modest, and some patients may become discouraged if early results are limited [148]. For this reason, a fiber-first approach should use realistic goal setting, for example, aiming for an initial weight loss of ∼5% to 10% of body weight, together with close follow-up [149]. If a fiber-first approach yields clinically meaningful weight loss and improvements in health markers, GLP-1RA therapy can be deferred or avoided. If the response is suboptimal, sequential addition of a GLP-1RA can promote further weight reduction [150]. In clinical practice, the WHO’s 2025 guideline conditionally recommends using GLP-1 therapies alongside intensive behavioral interventions, including structured dietary change and physical activity [151]. There is no strict rule for when to start combination therapy. Some clinicians wait ∼3 mo to assess the effect of diet and lifestyle changes, whereas others individualize timing based on patient motivation and engagement [152]. In practical terms, a fiber-first phase is low risk and low cost, it builds durable eating habits, and it can complement subsequent GLP-1RA therapy. However, in patients with substantial comorbidity burden, this approach may delay access to higher-efficacy options and should be weighed against clinical urgency [153].

**TABLE 3:** Integrated care pathways in obesity/overweight: GLP-1RAs × fiber

Path	Strategy (from Figure 6)	Suitable for	Advantages	Disadvantages
I	Dietary fibers only	Overweight (WHO standard, BMI 25–30 or ≥27 with mild risk); cost-sensitive; prefers nondrug; GLP-1RA contraindication/intolerance; maintenance after prior loss	Safe, inexpensive; metabolic and gut-immune benefits; good for long-term maintenance	Modest and slower loss; adherence-dependent; possible GI gas/bloating if titrated too fast
II	GLP-1RA → Fiber maintenance	Needs rapid initial loss (WHO standard, BMI ≥30 or ≥27 + comorbidity) and plans to come off drug (cost, AEs, and pregnancy planning)	Large early loss; fiber hand-over helps hold weight and reduce regain; lowers long-term drug exposure/cost	Regain risk if weak lifestyle base; GLP-1RA GI AEs; requires planned taper and follow-up
III	GLP-1RA only, then stop	Short-term targets (e.g., preoperative optimization) or therapeutic trial	Maximal short-term loss; quick risk reduction	High regain possibility after discontinuation without maintenance; drug cost/AE burden while on therapy; not durable alone
IV	Fiber first → add GLP-1RA	Step-up care; nondrug preference or budget limits; mild–moderate obesity where urgency is lower; escalate if response inadequate or risk increases	Builds diet foundation; low AE burden; cost-efficient; preserves GLP-1RA for when truly needed; can improve later adherence	Slower early loss; may delay needed pharmacotherapy, considering set objective review/escalation points
V	Concurrent GLP-1RA + Fiber	On GLP-1RA but seeks better satiety, regularity, diet quality; constipation management; intensive programs aiming for loss and durable habits	Loss ≈ GLP-1RA alone; fiber may improve tolerability (e.g., psyllium), fullness, micronutrient density, and future maintenance	Additive loss uncertain; regimen complexity; GI load with highly fermentable fibers; cost of both

This table summarizes conceptual care pathways integrating dietary fiber strategies and GLP-1RA therapy for obesity or overweight management. The listed pathways should be individualized according to clinical indication, treatment response, tolerability, cost, contraindications, and patient preference.

Abbreviations: AEs, adverse events; GI, gastrointestinal; GLP-1RA, glucagon-like peptide-1 receptor agonist.

A GLP-1RA–first approach is defensible in adults with obesity (>19 y, BMI ≥30) and severe or high-risk scenarios (BMI ≥40, or ≥35 with comorbidities) [154,155]. Initiating pharmacotherapy first does not obviate dietary intervention. Patients should still receive structured nutrition, physical activity, and behavioral support, because lifestyle change remains the foundation of weight management [156]. A major consideration with a GLP-1RA–first approach is follow-up after medication discontinuation. GLP-1RAs will not be feasible as lifelong therapy for all patients because of cost and adverse effects, and studies indicate that if therapy is stopped, a substantial portion of lost weight is typically regained, with a mean of about two-thirds within 1 y off treatment [20]. This is where a sequential strategy can be effective. After 1 to 2 y of GLP-1RA therapy, the dose can be gradually tapered and discontinued. At that point, a high fiber, nutrient-dense diet, for example, one rich in fruits and vegetables that serves as a proxy for overall diet quality, combined with regular exercise and behavioral support, can help support weight loss and limit weight regains [117,157]. In this framework, GLP-1RAs achieve the initial weight reduction, and dietary fiber becomes a cornerstone for long-term weight maintenance. This approach aligns with the chronic, relapsing nature of obesity care: an intensive early phase to reduce weight, followed by a maintenance phase focused on stabilizing outcomes and supporting metabolic health [115].

Taken together, irrespective of sequence, step-up or step-down strategies should be guided by the patient’s observed trajectory, including percentage weight change, tolerability, and the risk profile of obesity-related complications, given heterogeneity in fiber responses and variable persistence with GLP-1RA therapy in routine practice. A fiber-first strategy emphasizes safety, lower cost, and sustainable habit formation, although it may be insufficient for higher-risk patients. A GLP-1RA–first strategy prioritizes stronger short-term results, but it requires careful planning at discontinuation to sustain weight loss during maintenance. Using both in sequence is consistent with contemporary guidance and product labeling, which position pharmacotherapy within a comprehensive lifestyle framework.

In conclusion, GLP-1RAs have transformed obesity management, achieving robust weight loss, improving glycemic control, and conferring multiorgan benefits. Their efficacy, however, generally depends on sustained therapy because discontinuation frequently leads to weight regain. In contrast, dietary fibers act more gently and broadly, modulating the composition and function of the gut microbiota, strengthening the intestinal barrier, and stimulating endogenous GLP-1 secretion. Although weight loss with fiber is typically more modest when compared with GLP-1RAs, dietary fiber provides durable metabolic and immune benefits that support long-term health and may aid weight maintenance.

Given that both strategies converge on GLP-1 signaling and the gut–brain–immune axis, an integrated approach is attractive. Carefully selected NDCs may mitigate GLP-1RA–related GI adverse effects, improve bowel regularity, and help maintain satiety and microbiome stability when drug doses are reduced or discontinued. Conversely, GLP-1RAs may indirectly reshape the context in which dietary fibers act by delaying gastric emptying and altering fermentation kinetics.

Future research should prioritize well-designed trials that combine or sequence GLP-1RAs with clearly characterized dietary fiber types, including microbiome and immune readouts, besides the measurement of GLP-1R occupancy to clarify potential competition or synergy. As a conceptual model for future research and clinical testing, dietary fiber and lifestyle measures may form the foundation of care, whereas GLP-1RAs are used to achieve rapid weight reduction in high-risk patients, and fiber-supported microbiota resilience is leveraged to support long-term weight maintenance. By aligning pharmacotherapy with nutritional strategies, it may be possible to deliver more durable metabolic health while reducing costs, reducing adverse effects, and decreasing reliance on chronic medication.

At the same time, the potential cooperation between GLP-1RAs and dietary fibers warrants systematic investigation. We are now in a period where it is increasingly clear that the chemistry of fibers matters and can be fine-tuned for specific physiological outcomes. Soluble, viscous fibers such as psyllium may help counteract GLP-1RA–related constipation and support bowel regularity. Appropriate dietary fiber selection may help preserve microbial diversity and sustain SCFA production during GLP-1RA therapy, although it remains unclear whether this directly modifies drug-related changes in microbiota composition. These concepts remain largely hypothesis-generating, but they outline a promising direction for future research aimed at optimizing fiber type, dose, and timing to complement GLP-1RA therapy. Such work would advance precision nutrition in which dietary fiber chemistries are matched to individual patient needs to maximize synergy with pharmacological treatment.

Taken together, the future of obesity management will likely rely on rational combinations rather than single interventions. GLP-1RAs provide fast and substantial metabolic improvements, whereas dietary fibers offer a safe, accessible, and physiologically grounded strategy to sustain gut health, immune homeostasis, and long-term weight maintenance. The challenge and opportunity lie in mapping these interactions and harnessing their synergy. By combining the precision of pharmacology with the sustainability of nutrition, healthcare can move toward therapies that not only reduce body weight but also build metabolic resilience and improve overall health.

## Author contributions

The authors’ responsibilities were as follows – YW: contributed to conceptualization, investigation, writing – original draft, visualization; JL: contributed to investigation, writing – review & visualization; KV: contributed to validation, writing – review & editing; NGR: contributed to validation, writing– review & editing, visualization; RA: contributed to validation, writing – review & editing; and PdV: contributed to conceptualization, supervision, writing – review & editing.

## Data availability

Data described in the manuscript, codebook, and analytic code will be made available on request, pending application and approval.

## Declaration of Generative AI and AI-Assisted Technologies in the Writing Process

During the preparation of this work, the authors used ChatGPT (5.2) to correct grammar mistakes. The tool was not used to generate content. The authors then reviewed and edited the text as needed and take full responsibility for the content of the publication.

## Funding

YW is supported by the China Scholarship Council under Grant no. 202406780015.

## Conflict of interest

The authors report no conflicts of interest.
